# Global Challenges: Food, Agriculture and Nutrition

**DOI:** 10.1002/gch2.1005

**Published:** 2015-09-21

**Authors:** Spencer Henson

**Affiliations:** ^1^ Department of Food, Agricultural & Resource Economics University of Guelph Guelph Ontario N1G 2W1 Canada

## Abstract

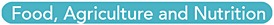

The broad complex of food, agriculture, and nutrition presents some of the greatest challenges facing the world today, not only in terms of their scale and consequences but also the difficulties faced in defining and implementing sustainable solutions. While many of these challenges are finally attracting commensurate attention across the public and private sectors, civil society, and research community, the related discourse is hampered by long‐standing economic, institutional, and political structures and interests. Furthermore, much of the research in this area fails to contend adequately with the complexity of the challenges presented by the global agri‐food system, reflecting an inability and/or unwillingness to work across disciplinary, epistemological, and/or methodological boundaries. No wonder, therefore, that there is growing urgency for policy‐relevant research that focuses on the most pressing global food, agriculture, and nutrition challenges and how these might be addressed effectively sustainably and at scale.

At the most fundamental level, the global agri‐food system is failing to ensure that an adequate and healthy diet is consumed by all. Currently, around 780 million people are undernourished, meaning that their intake of food is insufficient to meet their daily energy needs (FAO, 2015). Furthermore, billions of people are deficient in essential micronutrients; for example, 46% of women of childbearing age in South Asia are anemic (IFPRI, 2014). If achieving nutritionally adequate diets for all in the world today was not challenging enough, looking to the future there is the specter of ensuring that nine billion or more people have enough to eat while meeting growing demand for animal‐based foods as more and more people are lifted out of poverty. There is much debate over how to achieve this while not undermining the natural resource base and threatening environmental sustainability at the local and global levels, especially in the context of climate change.

While many in the world do not have enough to eat, many others are overnourished. Thus, we are observing increasing levels of obesity and a number of non‐communicable diseases (NCDs) that are related in part to a nutritionally poor diet. While NCDs were historically seen as a problem of the rich world, increasingly developing countries are subject to the double burden of undernutrition and overnutrition (IFPRI, 2014), imposing heavy costs on societies that are already economically weak. It is salutary to note that no country has ever been successful at reducing rates of obesity across its population (Roberto et al., 2015), indicating the inefficacies of prevailing strategies and highlighting the scale of the challenges needing to be faced.

Of course, the agri‐food system has broader and often significant socio‐economic and environmental impacts that reflect its natural resource base and sheer physical and economic scale, as well the inherent biological nature of food. Thus, there are concerns at the implications of the way in which the agri‐food system is organized and the actions of economic agents within it for the environment, exposure of populations to biological, chemical, and/or physical hazards, livelihood of the poor, welfare of animals, sustainability of rural communities, and so on. Identifying and measuring these impacts and defining viable ways in which they might be addressed are made difficult by the variety and complexity of agri‐food systems globally, and also the inherent economic and political interests that tend to sustain the status quo.

Evidently, the global agri‐food system faces enormous challenges that require actions across the public and private sectors and civil society at the international, national, and local levels. It is these challenges and the quest for practical and sustainable solutions that *Global Challenges* focuses on. Thus, the journal will provide a unique platform for the exchange of intellectual ideas and cutting‐edge research from across the natural and social sciences and humanities, with a specific focus on identifying consequences and possibilities for policy, relating to food, agriculture, and nutrition as well as more broadly.

Critically, *Global Challenges* aims to encourage and facilitate the publication of research that cuts across and indeed confronts established disciplinary, epistemological, and methodological boundaries in a manner that contends with the complexity of these challenges. We are looking to publish research that is not only of the highest quality but that also truly contributes to the definition and implementation of policy that addresses the most pressing challenges facing the world today. This might include innovative empirical analyses, systematic reviews of prior research, commentaries, and editorials. We are open to publishing work of the highest quality that addresses our vision of bringing real and significant change to the most pressing global challenges!
